# Implementation outcomes of a community dialogue intervention to improve primary care performance in a Ugandan rural health sub-district

**DOI:** 10.1080/16549716.2025.2541979

**Published:** 2025-08-07

**Authors:** Innocent Besigye, Robert Mash

**Affiliations:** a Division of Family Medicine and Primary Care, Faculty of Medicine and Health Sciences, Stellenbosch University, Cape Town, South Africa; b Department of Family Medicine, School of Medicine, Makerere University College of Health Sciences, Kampala, Uganda

**Keywords:** Community engagement, community dialogues, implementation science, processes, outcomes, implementation strategies, intervention evaluation

## Abstract

**Background:**

Since the declaration of Alma Ata, community participation in health services has been promoted in making services responsive to the needs of the people. This requires effective community engagement approaches. Community dialogues have been used to engage communities in design, implementation and evaluation of health activities and interventions.

**Objectives:**

This study evaluated the implementation outcomes of a community dialogue intervention that was intended to improve primary care performance in a health sub-district in rural Uganda.

**Methods:**

This was a mixed methods study using purposively selected key informants and a data collection form. The key informant interviews were conducted in English using a semi-structured interview guide, audio-taped and transcribed verbatim. Qualitative data was analysed using Atlas ti using a framework approach. Quantitative data was entered into an Excel spreadsheet and analysed into frequencies and percentages.

**Results:**

Overall, 196 community dialogues were conducted by all 16 primary care facilities, and the average attendance was 32 (range 16–46). They were found to be appropriate, acceptable and affordable and, therefore, adopted. They were feasible and implemented with fidelity, encountered minimal contextual barriers and were thought to be sustainable. Thirteen context factors enabled implementation (e.g. prior existence of regular outreach activities at each health facility), while two were barriers (e.g. community members’ expectations of incentives). The intervention reached all the health facilities within the health sub-district at no direct incremental cost.

**Conclusion:**

Community dialogues can be implemented through integration at no direct incremental cost and with significant reach to the population served with favourable outcomes.

## Paper Context

- **Main findings:** Community dialogues have favourable outcomes as an intervention for improving primary care performance.
- **Added knowledge:** Community dialogues can be implemented at no direct incremental cost through integration with the already existing health facility activities.
- **Global health impact for policy and action:** Community dialogues are an effective community engagement approach and can be used in addressing performance gaps in health services particularly in low resource settings.

## Background

Community participation has been promoted as one of the principles of primary health care (PHC) since the declaration of Alma Ata [1,2]. Involving the people in the planning, design and delivery of their health services improves responsiveness, relevance, equity and quality. It may also empower people and communities to improve their own health and the determinants of health. This has been re-affirmed in several global reports and declarations [3,4]. Such an intention requires a commitment to build community members’ capacity to make informed decisions as co-developers of their health services. The World Health Organization’s recently described PHC theory of change lists empowered people and communities as one of the three key components [5].

Communities can be instrumental in designing solutions to health problems through their involvement and dialogue [2]. Community engagement is increasingly being adopted in the design of interventions to improve health services. This increases their chances of success and sustainability. Community dialogue as a form of community engagement has been used in the promotion of public health interventions such as immunisations, pharmacovigilance and disease control programmes [6–8]. The use of community dialogues to improve PHC performance has not been routinely attempted despite the potential benefits.

Implementation of evidence-based interventions such as community dialogues requires evaluation to improve the design of the intervention, assess the implementation strategies, identify barriers and enablers to implementation and evaluate implementation outcomes. Such evaluations can guide further scale up and reduce waste of resources [9]. Typical implementation outcomes include the appropriateness and acceptability of the intervention, its feasibility, the fidelity to the original design, the costs, coverage and longer-term sustainability. Such outcomes have been evaluated in implementation of health care interventionsfor example, in provision of mental health care, disease prevention and management, as well as introduction of new technologies [6]. This study aimed to evaluate the implementation outcomes of a community dialogue intervention that was intended to improve continuity of care as a key domain of primary care performance in a health sub-district in rural Uganda.

## Methods

### Study design

This was a convergent mixed methods study using qualitative interviews and quantitative data collection tools to assess implementation outcomes. The implementation outcomes were as follows: adoption (uptake of the intervention by stakeholders), acceptability (how well the intervention was received by the stakeholders), feasibility (facilitators and barriers to implementation), appropriateness (the intervention’s fitness for purpose), fidelity (implementation as planned), coverage (number of communities and people reached by the intervention), cost (direct and indirect incremental costs) and sustainability (maintenance of the intervention over time).

The study was part of a broader study to demonstrate the utility of the Primary Care Assessment Tool (PCAT) in improving primary care performance in a district health system. Previous studies adapted the PCAT for use in Uganda [10], measured primary care performance, and used the evaluation of performance to prioritise and design community dialogues. Future studies may evaluate the effect of community dialogues on improving the utilisation and continuity of primary care

### Study setting

The study was conducted in the Mukujju health sub-district (HSD), one of the four sub-districts within the Tororo District. Tororo District is in Eastern Uganda, about 250 km from Kampala, the capital city. Mukujju HSD is located about 20 km from Tororo town. The HSD consists of one level IV (HCIV), nine level III (HCIII) and six level II (HCII) health centres. The HCIIs provide ambulatory primary care services, HCIIIs also provide maternity and obstetric care, while HCIVs also provide additional emergency obstetric and surgical care. Primary care is mainly provided by nurses, midwives and mid-level clinicians (clinical officers). Medical doctors are only available at the HCIV level. Health facilities conduct weekly outreaches where health providers move out to the communities for health education, immunisations and antenatal care. These activities are preplanned and are held on particular days of the week.

The population served by the HSD was rural with substance farming as the main economic activity. The population was predominantly young with a high school education and a high disease burden from infectious diseases. The majority seek care from the public sector facilities and cannot afford private health services.

### The intervention

The intervention was described as community dialogues and was co-designed with key stakeholders from the District Health Team (DHT), health facility managers, primary care providers and health unit management committee members. It was intended to improve utilisation and continuity of primary care. The intervention was implemented for 6 months using a community dialogue guide.

Implementation of community dialogues as a community engagement approach can be situated along the continuum proposed by Draper et al. ranging from mobilisation, collaboration and then empowerment [11]. For this study, community dialogues were conducted in communities within the health facilities catchment areas and integrated within the existing outreach activities to promote immunisations. This was done because of limited funding. The immunisation focal persons and nurses led the dialogues as part of the outreach. The Village Health Teams (VHTs) mobilised and informed community members to attend. Community dialogues were conducted with the community members before the actual outreach activities of health education, immunisation and antenatal care. Community dialogues consisted of the intervention implementation team describing to the community members the services offered by their nearest health facilities and the importance of seeking care from the same facility. The participants were then given opportunities to ask questions, make comments or seek clarifications concerning the services offered and the general operations of the facility. Therefore, the community dialogues used mobilisation, the lower level of community engagement and did not proceed to community collaboration and empowerment that occurs when all the specific steps that involve identification and exploration of the issue are followed by community-led generation of the solution.

### Implementation strategies

The strategies for implementing the intervention were categorised according to the implementation science typology and are described in Table 1 [12].

**Table 1.** Implementation processes, strategies and related implementation outcomes.

Implementation process	Specific implementation strategy	Strategy description	Implementation outcome(s)
Planning	Building district buy-in	The intervention design included members of the DHT as part of the stakeholders	Adoption and acceptability
Planning	Stakeholder engagement	Purposive selection of stakeholders (DHT, managers, primary care providers and HUMC members) to co-design the intervention in consideration of community needs	Appropriateness
Training	Training of intervention implementation team	Training of the outreach team to equip them with competences to conduct community dialogue using the guide	Feasibility
Quality	Developing a community dialogue guide	The community dialogue guide ensured uniformity in conducting community dialogues	Fidelity and feasibility
Training	Training intervention implementation	Training the implementation team on use of community dialogue guide	Fidelity
Planning	Identifying of resource persons in the community	The VHT members were identified for engagement as community members with experience in mobilisation of people for community health initiatives	Coverage
Integration	Intervention design and planning	Implementation of the intervention was embedded into the routine activities of the health facilities	Cost and sustainability

### Qualitative data collection

#### Study population

Evaluation of the intervention focused on the DHT members, health facility managers, primary care providers and HUMC members as key informants (KIs). They were included because they were either part of the group that designed or implemented the intervention.

#### Sampling procedure

The KIs were purposively selected to ensure representation of the different key stakeholder groups. A total of 18 key informant interviews (KIIs) were planned to include 2 DHT members, 4 health facility managers, 10 primary care providers and 2 HUMC members. Data collection continued until saturation was achieved at 10 interviews as determined by the last 4 interviews not yielding any new information.

#### Data collection

The KIIs were conducted in December 2023 using a semi-structured interview guide. The guide was developed by the first author (IB) from the design of the intervention and implementation strategies as well as the literature on community dialogues [13,14]. The guide started with an open-ended question seeking the opinion of the respondents on the implementation of community dialogues to improve ongoing care at health facilities. Then, the subsequent questions explored the implementation outcomes as listed in Table 1.

The KIIs were conducted in English by the first author (IB) and two trained research assistants. The interviews were conducted in private rooms at the health facilities. Each interview lasted 45–60 min and was recorded. Field notes were also taken during the interviews.

#### Data analysis

The KIIs were transcribed verbatim by an experienced transcriber. The transcripts were cross-checked with the recordings by the first author (IB). The transcripts were then uploaded into Atlas-ti for analysis using the framework method [15]:

1. Familiarisation: The first author (IB) read through the transcripts to familiarise himself with their content and in the process made notes on issues that could be coded.
2. Coding index: Codes were developed by the researchers (IB and RM) using the first two transcripts and grouped into categories.
3. Coding: Codes were then applied to all the transcripts.
4. Charting: Reports were created where all the data for specific categories were brought together.
5. Interpretation: The reports were then interpreted for themes using a deductive approach based on the implementation outcomes.

#### Trustworthiness

The lead researcher was a family physician with a background in qualitative research and mixed method designs. His passion for strong primary care may have influenced the process of data collection, analysis and interpretation. The lead researcher also has some relationship with the study HSD as it forms part of the Family Medicine teaching site under the department where he is employed. He was new to the concept of community dialogues and learnt with the stakeholders involved in the co-design of the intervention. Being aware of the need for reflexivity helped to minimise the effect of the relationship to the topic and HSD. Data collection and analysis were performed under the supervision of the second author (RM). The research assistants were employees of Tororo District as data clerks from the study HSD and may have been known to some of the respondents. However, they were trained on the objectives of the study and on how to use the KII guide during data collection.

### Quantitative data collection

Quantitative data included the coverage and cost of the intervention. Coverage consisted of the number of facilities that implemented the intervention, total number of community dialogues conducted during the implementation period and number of participants per dialogue. Cost estimations were not computed since no direct costs were incurred during the implementation. During the community dialogue sessions, data on coverage was collected using a structured form to capture relevant data. The people who attended each community dialogue session were counted by the team and recorded on the form. The data collected was entered into an Excel spreadsheet and analysed as frequencies, ranges and percentages.

### Ethical considerations

This study was approved by the Health Research Ethics Committee at Stellenbosch (S20/04/103) and the Makerere University School of Medicine Research and Ethics Committee (# REC REF 2020–164).

## Results

Ten people were interviewed, and their characteristics are shown in Table 2. They included two DHT members, two health facility managers, five primary care providers and one HUMC member. The respondents included primary care providers across all the facility levels in the HSD.

**Table 2.** Characteristics of key informants.

KII number	Gender	Age (years)	Health facility level	Role
I	Male	35	HCIII	DHT member
II	Male	32	DHO	Primary care provider
III	Female	29	HCII	Primary care provider
IV	Male	26	HCII	Primary care provider
V	Male	33	HCIII	HUMC member
VI	Male	48	HCII	Health facility manager
VII	Male	26	HCII	Primary care provider
VIII	Male	42	DHO	DHT member
IX	Male	41	HCIV	Health facility manager
X	Female	32	HCIV	Primary care provider

The themes are presented according to the implementation outcomes in the following subsections: 1) adoption, acceptability and appropriateness, 2) fidelity, feasibility and coverage and 3) cost and sustainability.

### Adoption, acceptability and appropriateness

A total of 196 community dialogues were conducted in all the 16 health facilities within the sub-district. The average attendance was 32 (range 16–46) people. These indicate that the intervention was acceptable and, therefore, adopted. The DHT members felt that the intervention had the potential to improve the utilisation of primary care services and could empower people to improve their health. The DHT members had been involved in the selection and design of the intervention and, therefore, found it easy to adopt. It is aligned with their goals to improve health and empowerment of communities:

We are trying whatever is possible to ensure that the communities own their health. (DHT member)

Most of the respondents acknowledged that community dialogues were an appropriate approach to improving health literacy and therefore the health and wellbeing of people. They all felt that the design of the intervention was appropriate and fit-for purpose:

For the few times we went to the community for dialogues and talking to them, we realised that there was a lot of ignorance. People are there in the community but they don’t know what happens, which services are offered in this level of health centre. (Primary care provider)

Interestingly, each of the stakeholder groups expected other stakeholder groups to automatically appreciate the relevance of implementing community dialogues to improve the quality of health services. For example, the DHT members expected the VHTs and other community leaders to naturally appreciate the role of community dialogues in health improvement and, therefore, their adoption and acceptability:

The Village Health Teams, the Local Councils, the opinion leaders should really appreciate the community dialogues. (DHT member)

According to the DHT members and primary care providers, the health facility managers were key in the adoption and implementation of the intervention. Their commitment, motivation and attitudes were instrumental as they were the decision-makers on what policies to implement and how to spend their budgets. All managers committed to the intervention and implementation but further engagement was done to strengthen their support. This engagement was done by the intervention implementers at the facilities discussing and explaining the intervention and its implementation with the facility managers:

We sat with the in-charges [health facility managers] themselves and agreed on certain things and ways they are to move forward and actualise [the intervention]. (Primary care provider)

### Fidelity, feasibility and coverage

The respondents did not report the need for any changes to the design of the intervention. The community dialogue facilitator guide and the training were helpful in ensuring fidelity to the design:

Yeah it was [intervention implementation] carried out as planned. (Primary care provider)

In terms of feasibility, participation was improved through the use of VHTs to mobilise and inform people about the dialogues. Therefore, the VHT members were vital to mobilising participation in their communities. Support from community leaders (cultural, religious and political) was also important as they were trusted by the communities. The respondents recommended use of media, for example, radio announcements to increase participation. The barriers to implementation were the distance of some communities from the health facilities, which made them harder to reach. Health facilities do not have vehicles to transport the outreach teams. They use public transport on bad roads and rough terrain. Community members also expected reimbursement for their time and travel in attending the dialogues. Most of the respondents mentioned that it is a common practice for external organisations and partners working with communities to provide such benefits from grants and donor funds. Such funds were not available in the public sector facility budgets:

Other organizations like there was a time PLAN International, the World Vision whenever like they would hold meetings they would come up with some refreshments. (Health facility manager)

The coverage of the intervention was good though some groups within the communities were harder to reach. For example, subsistence farmers were working on the land during the mornings when the dialogues were held and could not attend. Other groups, such as adolescents or older adults, might also have specific needs that might not be articulated in a dialogue open to everyone. Respondents also recommended better mobilisation of participants through community and social events, such as religious gatherings and burials (in churches or mosques) to improve coverage.

### Cost and sustainability

There were no incremental costs as the intervention was integrated into existing outreach activities for immunisation, health education and antenatal care. This approach also made the intervention sustainable:

We can still integrate with our activities at the facility like if we are going out to do immunisation, don’t only go and do immunisation where Uganda is going today, every opportunity you get, integrate. (Primary care provider)

Stakeholders emphasised that community dialogues should be conducted regularly from all health facilities and that they could easily become a routine activity. Although they were designed to discuss the utilisation of primary care facilities and the services available, other topics could be addressed in the future.

The respondents felt that even with the short period of implementation it had beneficial effects. The intervention was thought to have increased health literacy among the community members. Stakeholders perceived the intervention to have improved the community’s understanding of health care and healthcare delivery. The respondents also perceived the intervention to have strengthened the link between health facilities and the community. Generally, all the stakeholders had high hopes in the intervention with the view that it will greatly improve the health of the communities served over time:

It also helps to improve on service delivery at the facility, it also acts as a link between the community and the facility therefore I recommend it to continue. (DHT member)

## Discussion

### Summary of the key findings

**Figure 1.**
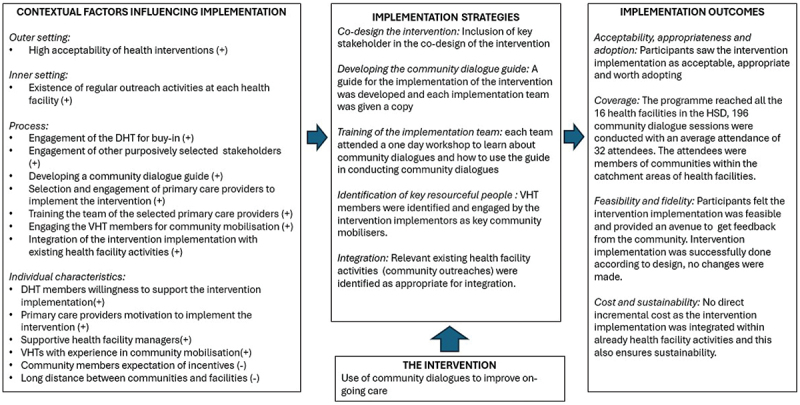
Summary of intervention implementation strategies and outcomes.

The key findings with regard to the implementation outcomes and contextual factors are summarised in Figure 1 as an implementation research logic model [16]. Figure 1 also includes the implementation strategies and intervention as outlined in the methods to give a complete overview of implementation. Community dialogues were adopted and found to be appropriate, acceptable and affordable in this low resource setting. They were feasible to implement according to the original design and met with very few contextual barriers. Minor barriers included the distance and difficulty with travel to some remote communities and the expectation of reimbursement from community members for their participation. Participation was enabled by the VHTs and community leaders. Community dialogues were seen as a sustainable approach to community engagement which could address a variety of issues in the future.

### Discussion of the key findings

Studies on the implementation of community dialogues in Uganda, Mozambique and Zambia also found them acceptable and appropriate to improve uptake of childhood health programmes and disease prevention [6,7]. These studies also reported that community dialogues were adopted by both stakeholders and communities. Community dialogues were also found to be an acceptable intervention in patient empowerment studies [8,17].

In almost all studies evaluating the implementation of community dialogues, the mobilisation role of community health workers and community leaders is highlighted as key to their feasibility. In this study, the VHT members played a great role in the mobilisation of participants and did so without any payment or incentives. Voluntary participation in mobilisation for community dialogues by CHWs has also been reported in Uganda and Mozambique, while in Zambia they were paid a stipend for motivation [7].

The intervention had good coverage across the whole HSD. The average attendance of community dialogues was similar to what has been reported elsewhere as well as the need for timing of the dialogues on particular days or times of the day in accordance with the local context [8]. This is in line with the findings from our study where it was reported that subsistence farmers had difficulty attending the sessions as they were busy working on their allotments. Several other studies report the use of media, such as radio and short message services (SMS) to increase attendance of community dialogues [8,17]. This was also recommended by the study participants. However, this could not be done in our study as it required direct funding that was not available. The intervention was co-designed with the aim of improving continuity of care as the identified primary care performance gap, but the actual implementation focused on improving utilisation as a prerequisite for improving continuity [18].

The cost of the intervention was offset by embedding it within the already existing health facility outreach activities. Such an approach was also used to implement community dialogues in Bangladesh [19]. This approach could ensure sustainability of the intervention and enable engagement of the community on other health issues in the future.

There were few barriers to implementation, although distant communities were difficult to reach due to geographical and logistical challenges. A scoping review of the Organisation for Economic Cooperation and Development member countries concluded that distance was a factor associated with limited access and use of services particularly in primary care services [20]. Other studies have also documented poorer health outcomes associated with longer distances from health facilities [21].

Community dialogues provide an opportunity to listen and get feedback from the communities served. This depends on the ability and willingness of the implementers to receive this feedback and act on it to improve the health services. The feedback received can also be used in modifying the implementation strategies to align with the unique characteristics and life conditions of the involved communities. This can improve the appropriateness, acceptability, coverage and sustainability of the intervention. In this study, community dialogue participants recommended ways to improve feasibility and coverage. Other studies have documented that community dialogues lead to recommendations to improve the health services and care of patients [8].

## Strengths and weaknesses

The mixed methods explored and evaluated all the implementation outcomes from multiple perspectives, although evaluation of sustainability requires longer follow-up beyond the 6-months in this study. All stakeholder groups were included, although the voices of the community members and VHTs could have been stronger and might have added additional perspectives. Evaluation of coverage could have included more detail on gender, age and characteristics to determine which sections of the community participated. Nevertheless, the findings may be transferable to other districts in similar low resourced and rural settings. The qualitative component of the evaluation included only those who had either participated in the design or implementation of the intervention. Therefore, the evaluation may have missed the relevant views of those who were not involved in the design and implementation of the intervention.

## Implications

Community dialogues are a suitable approach to community engagement in low resource settings such as this rural health sub-district in Uganda. They were seen as appropriate and acceptable and were feasible to implement with good fidelity and minimal cost. Facility managers were key to successful implementation. Committing some of the budget to community engagement might overcome some of the barriers to participation. Skills in conducting community dialogues can be integrated into the training of primary care providers. Further research is needed to determine if utilisation or continuity improves and the PCAT can be used to measure this. Results can be compared to the same sub-district at baseline or to neighbouring sub-districts that did not have this intervention.

## Conclusions

The implementation of community dialogues to improve continuity of care as a key domain of primary care performance was adopted within the HSD and was acceptable, appropriate, feasible and sustainable. They can be implemented through integration with existing activities, at no direct incremental cost and with significant reach to the population served. Therefore, community dialogues are an effective community engagement approach in low income and resource-constrained settings.
